# Strategies for evidence-based in head and neck cancer: practical examples in developing systematic review questions

**DOI:** 10.3389/froh.2024.1350535

**Published:** 2024-02-02

**Authors:** Eliete Neves Silva Guerra, Juliana Amorim dos Santos, Ricardo D. Coletta, Graziela De Luca Canto

**Affiliations:** ^1^ Laboratory of Oral Histopathology, Health Sciences Faculty, University of Brasilia, Brasilia, Brazil; ^2^ Department of Oral Diagnosis and Graduate Program in Oral Biology, School of Dentistry, University of Campinas, Piracicaba, Brazil; ^3^ Brazilian Centre for Evidence-Based Research, Department of Dentistry, Federal University of Santa Catarina, Florianópolis, Brazil

**Keywords:** education and practice, evidence-based, systematic review, meta-analysis, head and neck cancer

## Abstract

A systematic review (SR) requires several steps to be conducted. A major and initial challenge is to formulate a focused research question that may have high scientific relevance to provide evidence-based results and strategies. This narrative mini-review aims to present different categories of systematic reviews currently applied in Head and Neck Cancers (HNC), focusing on the strategies to provide results for evidence-based decision making. The SRs identified were of intervention, diagnostic testing, prognosis, *in vitro* and *in vivo* studies, prevalence, and epidemiological studies, and of association and risk factors. Focused questions that define the type of review, whether it is a therapy question (intervention), a question of prevalence or an outcome (prognosis) of disease, are discussed. Additionally, the importance in building interesting research questions and following all proposed steps to produce quality evidence are highlighted. This narrative mini-review may guide future research by showing how to perform and report relevant evidence in terms of HNC.

## 1 Introduction

Head and Neck Cancer (HNC) is one of the most prevalent malignancies affecting diverse regions of the oral cavity, larynx, and oropharynx (1). In 2020, the last GLOBOCAN estimative underscored over 714,000 cases of HNC worldwide. Among these, Oral Squamous Cell Carcinoma (SCC) emerges as the most common subtype accounting for 377,713. The anticipated estimative for 2025 predicts a continued rise in HNC cases, reaching over 800,000 cases globally. Notably, 52% of these cases are expected to occur in the oral cavity (2).

The development of HNC involves a complex series of events. Its progression is marked by a gradual genetic and epigenetic variations affecting cell growth, survival, and microenvironment interactions (3). It this context, lifestyle is highlight as a major point. Different forms of tobacco use, including betel quid/areca nut, alcohol consumption, and human papillomavirus (HPV) infection are the most related risk factors (4). Tobacco and alcohol, whether separately or in combination, are mainly associated with oral SCC. Additionally, HPV infection, particularly HPV type 16, has been associated with oropharyngeal, and ultraviolet radiation sunlight exposure with lip SCC (5, 6).

**Figure 1:**
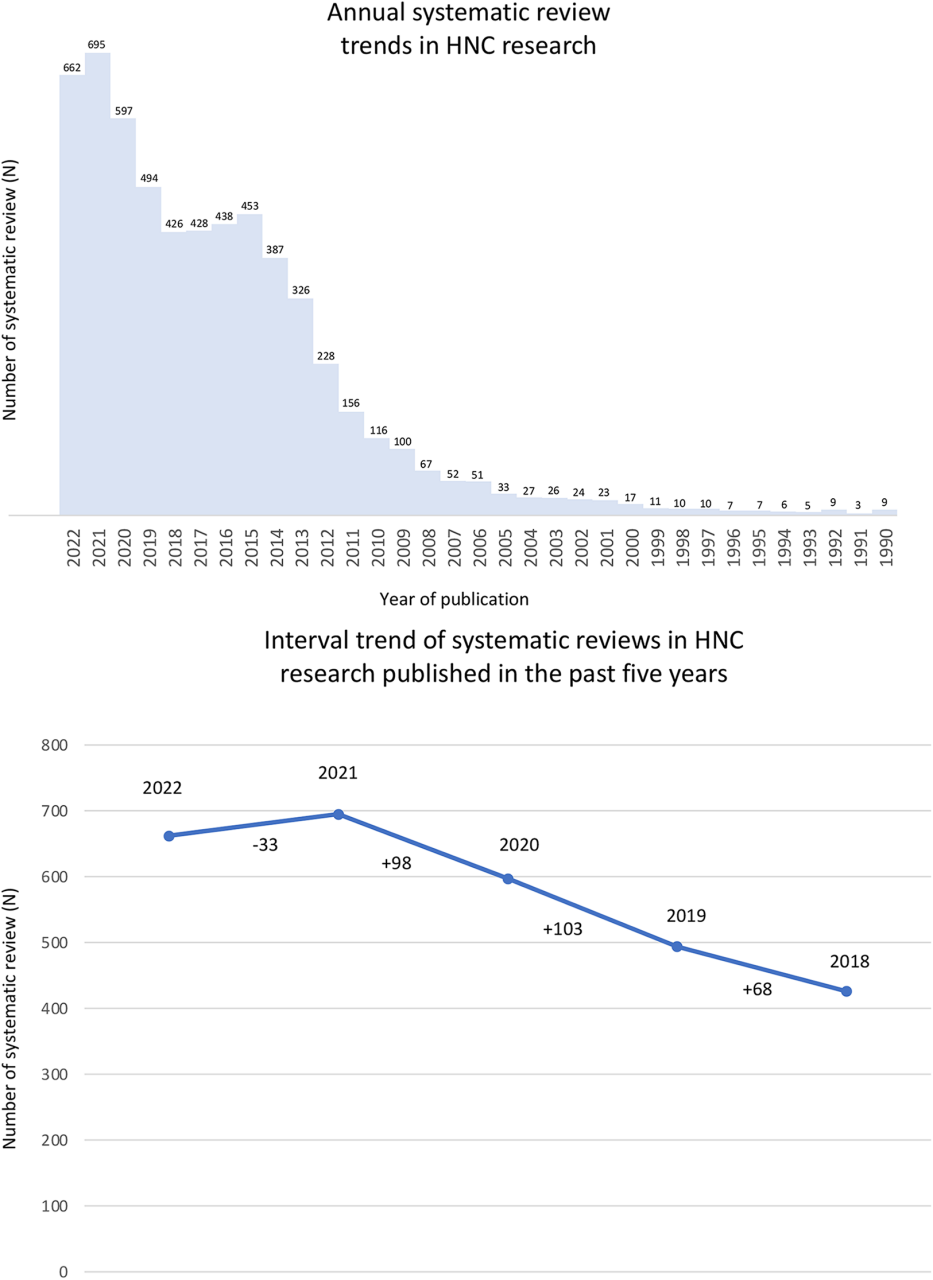
The number of systematic reviews in head and neck cancer research (PubMed search until 2022). (**A**) A rapid search using “mouth neoplasms” (MeSH Terms) OR “head and neck neoplasms” (MeSH Terms) terms were performed on PubMed to analyze the annual systematic reviews trend in Head and Neck Cancer research (filter applied: “systematic review” OR “meta-analysis”). (**B**) Trend difference of systematic reviews in Head and Neck Cancer research published in the past five years.

Considering the importance of HNC, the number of Systematic Reviews (SR) has increased over the years (Figure 1A). In 2022, 662 SRs were dedicated to different aspects of HNC, with the trend difference reaching more than 100 SRs between 2019 and 2020 following PUBMED database search (Figure 1B). Nonetheless, conducting a SR requires experience in translating clinical issues to a research question considering its maximum efficiency to find relevant evidence. This experience improves the ability to critically evaluate the evidence and apply the results in clinical practice. According to the Preferred Reporting Items for Systematic Reviews and Meta-Analyses (PRISMA) statement, a SR requires several steps, such as defining a focused question, establishing inclusion and exclusion criteria, searching databases, selecting studies, appraising methodological quality or risk of bias, collect data, and synthesize results (7).

To manage a SR, researchers must be prepared to identify relevant issues that require information synthesis and develop adequate questions to accurately assess the evidence. Thus, the purpose of this article is to present the main principles of methodologies applied to different SR categories on HNC. This narrative mini-review based on authors' experience guides the formulation of questions according to the acronym's strategies for SR.

## 2 Types of systematic reviews in HNC

### 2.1 SR of intervention

Interventional SR is a classic and well-known type of review that aims to assess and compare treatment options. The purpose is to summarize evidence regarding the effects of health care or social interventions for certain diseases or conditions. Ideally, it should be performed including randomized clinical trials (RCT) because of its high level of evidence. If there is a lack of RCT, non-RCT studies or observational studies can be included, however the level of evidence is lowered (8).

To illustrate the strategy for a SR of intervention, we can cite the article by Normando et al. (9) which aimed to assess the effects of turmeric and curcumin for oral mucositis. The authors used the acronyms PICOS with detailed explanation to formulate a precise research question (Table 1). Applying this strategy, the study showed strong evidence for the application of turmeric and curcumin in the treatment of chemo/radio induced oral mucositis. Both turmeric and curcumin were able to reduce pain, erythema intensity, ulceration area, and degree of severity of oral mucositis. They were also effective in prevention by delaying the onset of the lesions. However, the authors suggest further investigation to improve and confirm de evidence.

**Table 1:** PICOS strategy to develop a focused question for SR of intervention.

Main HNC concern	Chemo/radiotherapy induced oral mucositis in HNC
PICOS strategy for SR of intervention	
Participants or population	Cancer patients undergoing chemotherapy and/or radiotherapy
Intervention	Turmeric or curcumin
Comparison or control	Placebo or other interventions
Outcomes	Prevention or treatment of oral mucositis
Studies	Clinical trials (randomized or not)
Final research question	Is there any effect of turmeric and curcumin in the management of oral mucositis induced by chemotherapy and/or radiotherapy in cancer patients?

### 2.2 SR of diagnostic testing

Diagnostic tests are used by health professionals to discriminate whether an individual has a particular disease or condition in populations considered to be suspect for the disease (10). It is known that a test is sensitive when it can discriminate among suspects, those who are effectively ill. Sensitivity is the reason of the number of true positive assessments per number of all positive assessments. Differently, specificity is the ability of the same test to be negative, which represents the reason of the number of true negative assessments per number of all negative assessments (11). The SR of diagnostic test allows investigating the validity of an index test compared to a reference test (reference standard), considering different designs of diagnostic studies and different population profiles (10). Thus, the SR of diagnosis is a very important tool to study the applications of new exams and diagnostic methods, taking in consideration accuracy, sensitivity, and specificity measures.

As an example, Guerra et al. (12) aimed to assess the capability of serum biomarkers to diagnose HNC. For this purpose, the acronyms PIRDS were applied with detailed explanations (Table 2). The study was able to demonstrate promising serum biomarkers in the diagnosis of HNC. Accuracy was improved by the combination of EGFR + Cyclin D1 and SCCA + EGFR + Cyclin D1. The results have also shown higher sensitivity and specificity when compared to isolated biomarkers. The authors pointed out the need for further well-structured research to validate these biomarkers.

**Table 2:** PIRDS strategy to develop a focused question for SR of diagnostic testing.

Main HNC concern	Biomarkers for diagnosis of the HNC
PIRDS strategy for SR of diagnostic testing	
Participants or population	Individuals with HNC
Index test	Serum (blood) biomarkers
Reference test	Biopsy followed by histopathological analysis
Diagnosis of interest	Accuracy, sensitivity, and specificity measures
Studies	Diagnostic testing studies
Final research question	Do serum (blood) biomarkers have the capability to accurately identify HNC patients from non-HNC controls?

### 2.3 SR of prognosis

The SR of prognosis is an excellent tool and widely used in HSC field as it is the most adequate review to summarize overall outcomes. It is also used to determine the importance of an exam to determine the prognosis and to identify prognostic factors and predictors of an individual's response to treatment, associated or not with changes in health outcomes (13).

Illustrating this type of SR, Dourado et al. (14) evaluated the impact of cancer-associated fibroblasts (CAF) on oral cancer prognosis. The focused question was structured using a PICOS strategy with detailed information (Table 3). Based on that, authors (14) found CAF as an appropriate prognostic biomarker and therapeutic target in oral cancer. The high expression of CAF was associated with worse overall survival and disease-free survival in oral cancer. Moreover, a correlation of the abundance of CAF and the clinicopathological features could be suggested reflecting in aggressiveness and dissemination of this disease.

**Table 3:** PICOS strategy to develop a focused question for SR of prognosis.

Main HNC concern	Prognostic factors associated with survival in HNC
PICOS strategy for SR of Prognosis	
Participants or population	Individuals with oral cancer
Intervention or exposure	CAF analysis by immunohistochemical detection with anti-α-SMA antibody
Comparison or control	Normal tissue (oral mucosa)
Outcomes	Overall survival and disease-free survival
Studies	Observational studies in humans
Final research question	Do immunodetection of cancer-associated fibroblasts (α-SMA-positive fibroblasts) serves as a prognostic factor of the survival of patients with oral cancer?

CAF, cancer-associated fibroblasts; α-SMA, alpha smooth muscle actine.

### 2.4 SR of *in vitro* and *in vivo* studies

*In vitro* experiments with cell culture and *in vivo* animal models are studies widely used in the research routine of pathology and histology laboratories. SR with these types of studies provide an excellent source for identifying gaps in the translational research, gaining knowledge and ideas to improve laboratory questions. However, it should be noted that SR of *in vitro* and *in vivo* animal studies present a lower level of evidence compared to clinical and observational studies (15). Although there are limitations associated to *in vitro* and *in vivo* SR, it is often necessary to answer about specific topics or when there is no stronger evidence available.

Aiming to assess curcumin as an alternative treatment for HNC, Borges et al. (16) conducted a SR with *in vitro* and *in vivo* studies. Note that the decision to incorporate laboratory studies was driven by the recognition of the lack of evidence on this topic in a clinical context. The focused question was based on a PICOS acronyms (Table 4). In this study, the authors found curcumin as an effective inhibitor of proliferation and survival in HNC cells. The SR also demonstrated its effects on reducing tumor measurements in animal models. While this SR provides an initial level of evidence, it reinforces the potential of curcumin as an adjuvant drug in HNC treatment. The immediate application of these findings to patients may not be feasible, but it supports the initiation of clinical trials based on fundamental evidence.

**Table 4:** PICOS strategy to develop a focused question for SR of laboratorial studies.

Main HNC concern	Cellular response and tumor size in HNC
PICOS strategy for SR of laboratorial studies	
Participants or population	Cell cultures (*in vitro*) and animals (*in vivo*).
Intervention or exposure	Curcumin
Comparison or control	Untreated, placebo or substances other than curcumin
Outcomes	(i) *in vitro* cell proliferation, viability or cytotoxicity or *in vivo* tumor volume or tumor incidence and (ii) apoptosis and/or cell cycle arrest, including analysis of protein expression.
Studies	Experimental *in vitro* or *in vivo* animal studies
Final research question	What are the *in vitro* effects of curcumin on the proliferation and survival of head and neck squamous cell carcinoma cell cultures and the animal *in vivo* effect on tumor size?

### 2.5 SR of epidemiology studies

SR from epidemiological studies is necessary to regulate health conditions trends in terms of prevalence and incidence. This type of SR can also determine the frequency of clinical, radiographic, or histological findings as signals or symptoms of certain diseases. Data synthesis provided by this type of review is a powerful tool to inform social and healthcare professionals, policymakers, and consumers on the decisions-making moment (17).

As an example of SR of prevalence in HNC, we can cite the article by Moura et al. (18). This review serves as a model for frequency surveys regarding pathway mutations. It aimed to define the prevalence of PI3K-AKT-mTOR signaling pathway mutations in patients with HNC. Using the PEOS strategy to formulation a focused question (Table 5), the authors found an estimated mutations prevalence ranging from 2% (*AKT*) to 13% (*PIK3CA)* for the related genes. To make the evidence more robust, they could also perform subgroup analysis according to risk factors and tumor characteristics, including HPV infection, tobacco use, alcohol exposure, TNM stage, and histological tumor differentiation. Moreover, the findings demonstrated that PI3K-AKT-mTOR pathway emerges as a potential prognostic factor and could offer a molecular basis for future studies on therapeutic targeting in HNC patients.

**Table 5:** PEOS strategy to develop a focused question for SR of epidemiology studies.

Main HNC concern	Prevalence of gene mutations in HNC
PEOS strategy for SR of prevalence and epidemiology studies	
Participants or population	Individuals with HNC
Exposure	Mutations in the following genes of the PI3K-AKT-mTOR signaling pathway—PIK3CA, AKT, MTOR, and PTEN
Outcomes	Prevalence of mutations in the PI3K-AKT-mTOR pathway
Studies	Observational studies and clinical trials (randomized and non-randomized).
Final research question	What is the worldwide prevalence of PI3K-AKT-mTOR pathway mutations in head and neck cancer?

### 2.6 SR of association and risk factors

SR of association and risk factors assess individuals' characteristics or habits, such as genetic aspects or environmental exposure, and the risk of developing health conditions. The risk factors can be modifiable, for example, cigarette smoke, or non-modifiable – family history (19). Therefore, SR in this field presents important evidence that influences health practice, not only for professional's care decisions, but also for population counselling.

The SR published by Mello et al. (20) is a model of association review using the PECOS strategy (Table 6). This study aimed to answer if there is an association between mate consumption and the occurrence of upper aerodigestive tract (UADT) cancer. As results, they found an increased chance of cancer occurrence in all UADT subsites (oral, pharynx, esophagus, and larynx) when mate consumption was present. Secondary outcomes showed that high volumes of mate consumption per day increased odds of developing UADT; however, the temperature of consumption did not impact its occurrence.

**Table 6:** PECOS strategy to develop a focused question for SR of association and risk factors.

Main HNC concern	Association and risk factors for HNC
PECOS strategy for SR of association and risk factors studies	
Participants or population	Humans
Exposure	Mate consumption
Comparison or control	No consumption
Outcomes	Association with the occurrence of UADT cancer
Studies	Observational studies (cohort, case-control and cross-sectional)
Final research question	Is there an association between mate consumption and occurrence of UADT cancer?

UATD, upper aerodigestive tract.

## 3 Final considerations

SRs and meta-analyses provide huge benefits for human health by contributing to the evidence-based practice, which reduces the gap between research findings and health care practice (21). This type of scientific reports systematically summarizes and critically appraises available evidence regarding specific topics on health fields, resulting in qualified evidence or suggesting future research needs, when data is limited or non-existent (22). However, the increasing number of published SRs indicates the need to systematize even more the scientific production process (23). In some cases, although methods of SRs and meta-analysis were well developed, the published evidence is not updated, leading to an inability in maintaining its relevancy and accuracy (21). Concerned about the research commitment on updating reviews with a predetermined frequency, Elliot and collaborators (21, 24) have suggested a new approach to add in standard SR methods, named living SR. Living SR can be applied to any type of review, and it consists of continued surveillance for new evidence to include relevant information into reviews already published, remaining evidence always being updated (21, 24).

Since results obtained in SRs may influence healthcare and research decisions, minimizing risks of error and bias is fundamental (22). In this context, we highlight the importance of following all proposed steps to produce quality evidence. Research should begin from the protocol formulation, containing a PICOS (or similar) strategy designed to answer a focused question, which will establish well-defined inclusion/exclusion criteria, and should assess the quality of the evidence provided without missing any steps (7). We also encourage researchers to practice the living SR as a part of the publication protocol. It will help to incorporate relevant new evidence as it becomes available, allowing the opportunity to narrow the evidence-practice gap (24).

This narrative mini-review presents some limitations. Firstly, the content was focused on methodology applications not on reporting specific data results about HNC. Secondly, the systematic reviews included for discussion were selected according to authors experience with these types of studies. Despite that, it summarizes the different types of SR applied in HNC exploring adequate strategies to provide and report results focus on evidence-based decision making.
